# Fever, Headache, and Visual Blurring in a 17-Year-Old Woman

**DOI:** 10.1371/journal.pmed.0010007

**Published:** 2004-10-19

**Authors:** William Lynn, Sue Lightman

## Abstract

A fascinating case, with much to learn about diagnosis and treatment of patients with abnormal CSF results. After learning from the case, take our online quiz

## DESCRIPTION of CASE

A 17-year-old woman, who was born in Bangladesh, presented to an accident and emergency department in the United Kingdom with a history of being unwell for 24 hours. She had a headache and fever, and was vomiting. On questioning, there was no photophobia or neck stiffness. She lives in the United Kingdom and had been for a holiday to Bangladesh 4 months previously. The family members were all well, and none had similar symptoms. The woman had no previous medical history and was on no medication.

On examination, she looked unwell, with a temperature of 38.5 °C, pulse 96 beats per minute, blood pressure 112/52 mm Hg, and a respiratory rate of 16 breaths per minute. She had a Glasgow Coma Score of 14, there was no neck stiffness, and her ocular fundi were said to be normal. There were no other significant findings on examination. Immediate investigations showed a normal blood count apart from a raised white blood cell (WBC) count of 11.1 × 10^9^ per millilitre (normal range, 4.3–10.8 × 10^9^, per millilitre), with 84% neutrophils. Her erythrocyte sedimentation rate was 15 mm in the first hour (normal range, 1–12 mm), and her C-reactive protein was less than 5 mg/l (normal range, less than 10 mg/l). A malaria screen was negative, and renal and liver function tests were normal.

### What Clinical Diagnoses Were Being Considered?

A clinical diagnosis of probable viral meningitis was made pending the results of further investigations. Chest X ray was normal, as was an unenhanced CT brain scan. Lumbar puncture showed a normal cerebrospinal fluid (CSF) opening pressure, and the CSF was not blood stained. Laboratory analysis showed 1,606 WBC/ml (normally there are less than 5 cells/ml), of which 60% were lymphocytes with protein of 1.08 g/l (normally less than 0.6 g/l) and glucose of 2.9 mmol/l (normally greater than 50% of plasma glucose), with a corresponding plasma glucose of 5.7 mmol/l (normal range, 4–9 mmol/l). No organisms were seen on Gram stain. The CSF was negative for pneumococcal, meningococcal, and Haemophilus antigens, and bacterial culture was subsequently negative. The patient was given 2 g of ceftriaxone intravenously immediately, and ceftriaxone treatment was continued following the CSF results. Aciclovir IV, at a dose of 10 mg/kg every 8 hours, was added to cover the possibility of herpes encephalitis.

### What Was the Subsequent Differential Diagnosis?

The differential diagnosis was now viral, bacterial, tuberculous (TB), fungal, or malignant meningitis, or sarcoidosis. Additional investigations were requested, including polymerase chain reaction (PCR) on CSF for viruses and tuberculosis. There were no risk factors for HIV infection (HIV seroconversion can present with meningitis). Mollaret’s meningitis (recurrent aseptic meningitis associated with herpes simplex virus) was a possibility [1], though this condition characteristically presents as *recurrent* episodes of apparent aseptic meningitis.

The following morning the patient’s temperature returned to normal at 37 °C, and she felt better. Referral was made to the Infectious Disease Service. Twenty-four hours after admission, she was afebrile but clinically worse, with marked headache and vomiting. A day later she spiked a fever of 39 °C. No organisms were cultured from the CSF, urine, or blood. CSF bacterial antigens, cryptococcal antigen, and CSF auramine stain (for mycobacteria) were also all negative.

The following day the patient complained of further headache, nausea, blurred vision, and photophobia. In addition, she was noted to have bilateral large pupils, which did not react to light, and very pink optic nerves. Papilloedema was thought likely, and no other neurological signs were detected. The original CSF sample was negative on PCR for tuberculosis and herpes simplex virus, C-reactive protein remained at less than 5 mg/l, and a further head CT scan with contrast was normal.

The diagnosis was revised to meningoencephalitis. Other agents—such as listeria, tuberculosis (a systematic review found that PCR has a sensitivity of only 56% [95% CI, 46%–66%] for detecting TB meningitis [2]), and viruses such as others in the herpes group, mumps, and West Nile virus—were considered. The aciclovir was stopped, and quadruple tuberculosis therapy was started.

### What Did the Eye Signs Mean?

**Figure 1:**
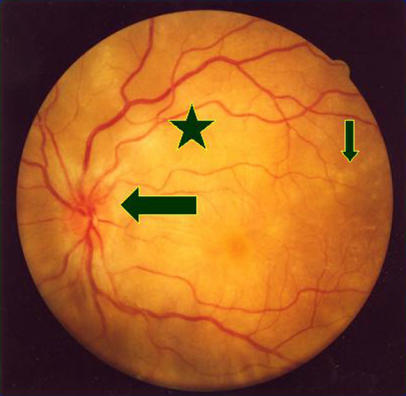
Fundal Appearance of the Patient's Eye The large arrow indicates the pink optic nerves; the star shows localised retinal detachment; and the small arrow pointing down shows small, white choroidal granulomas.

The fixed, dilated pupils were of major concern, and urgent ophthalmic review was requested. Examination by the ophthalmologist showed reduced vision at 6/60 right eye and 6/36 left eye. On testing with Ishihara charts, the patient had severely reduced colour vision. Her pupils were large and non-reactive to light or accommodation in both eyes. The eyes were inflamed, with cells present in the aqueous and vitreous humours. The optic nerves were swollen and very pink (Figure 1), but there was normal spontaneous venous pulsation present—demonstrating that this was not papilloedema (see Video 1). Bilateral choroidal infiltrates with overlying serous retinal detachments were also present.

**Video 1:** Spontaneous Venous Pulsation of the Veins at the Optic Nerve Head

### What Was the Final Diagnosis and Treatment?

The combination of the clinical symptoms, signs, and ocular features was characteristic of Vogt-Koyanagi-Harada (VKH) syndrome [3]. All antibiotics were stopped, and high-dose corticosteroids were started at 100 mg prednisolone daily. At one week there was no significant ocular improvement, although the patient's headache and vomiting were now gone, and she felt much better. Additional immunosuppressive therapy was initiated with cyclosporin and mycophenolate, and within a further week the patient's vision started to improve, with settling of the ocular signs. Oral corticosteroids were tapered, as was the cyclosporin, and by one month the patient's vision had returned to normal, and the ocular signs continued to settle. By three months the cyclosporin was discontinued, the steroid dose was reduced to 5 mg daily, and the mycophenolate dose was tapered. By six months all therapy was discontinued. At review six months later, the patient remained well, with normal vision and normal optic nerves.

## DISCUSSION

This young patient presented acutely with a fever and some signs suggestive of meningitis. She was initially treated as having viral meningitis, but the CSF findings indicated that other aetiologies needed to be considered. In particular, in a woman who previously lived in and recently visited Bangladesh, with a lymphocytic meningitis and borderline CSF glucose, tuberculosis had to be considered. Initially the ocular symptoms and signs were not a prominent feature, but the signs were likely to have been present when the patient was first seen.

The typical CSF changes associated with meningitis of different aetiologies are shown in Table 1. In this case the mixed lymphocytes and neutrophil leucocytosis with a borderline CSF glucose on the patient's initial CSF sample were consistent with bacterial or TB meningitis. Viral infection was far less likely, as only mumps is consistently associated with reduced CSF glucose.

**Table 1:**
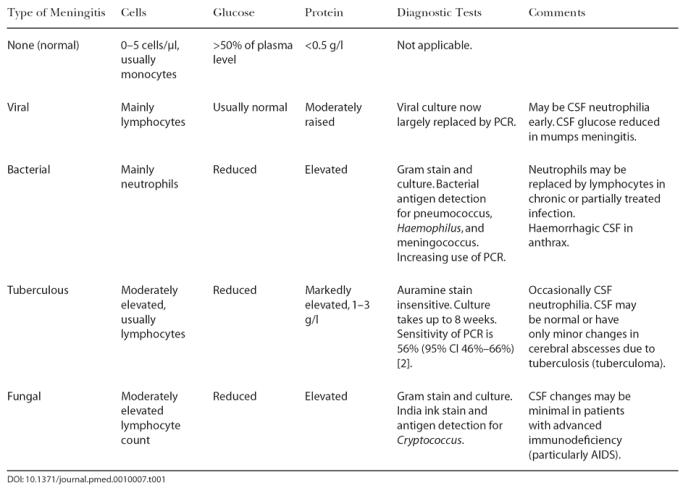
CSF Changes in the Most Commonly Encountered Types of Meningitis

### Infectious Causes of Meningitis

There is a wide range of infectious causes of meningitis worldwide. The likely infecting organism will be determined by the age and immune status of the patient plus the situation in which the infection was contracted. Thus, in an immunocompetent adult in the UK, enteroviruses are the commonest cause of viral meningitis, with meningococcus and pneumococcus the commonest bacterial agents. Tuberculosis is more common in people who have lived in a highly endemic area. In other parts of the world, the differential diagnosis may include viral infections such as West Nile virus in the continental United States and Japanese B encephalitis in Asia, or other pathogens such as rickettsiae, borrelia (Lyme disease), and protozoa. In the immunocompromised host, listeria must be considered, and there is an increased risk of fungal infection and tuberculosis. Finally, it is important to consider sexual exposure, as both secondary syphilis and HIV seroconversion may present with meningitis.

It is important, therefore, in the evaluation and management of patients presenting with a meningoencephalitis that the differential diagnosis be continually reviewed if the patient is not responding to therapy (Table 2). When appropriate investigations have been performed and are negative and symptoms persist, non-infective causes of CSF inflammation must be considered—as turned out to be the case here (Table 3).

**Table 2:**
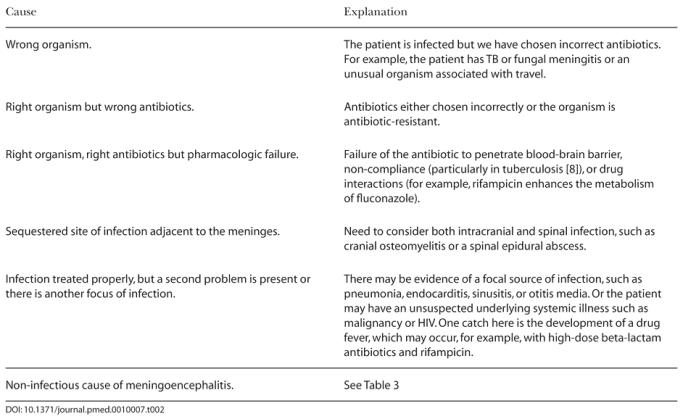
What to Do When the Patient Is Not Getting Better

**Table 3:**
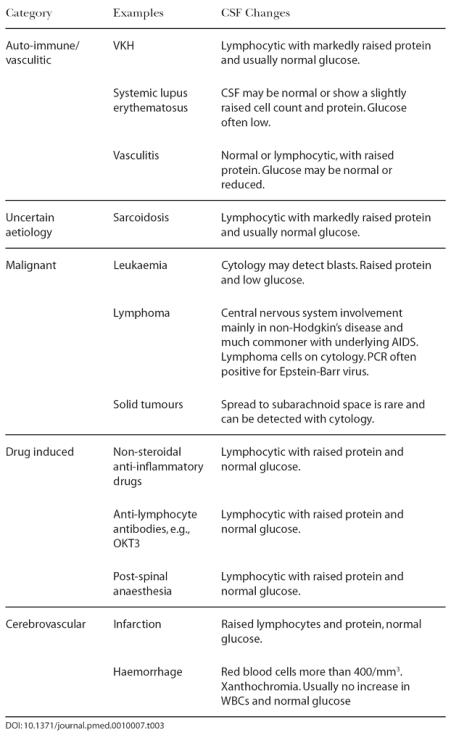
Non-Infectious Causes of Abnormal CSF

### VKH Syndrome

VKH syndrome [4,5,6] is a systemic disease involving various melanocyte-containing organs. It is rare in white Northern Europeans and white Americans but much more common in people with darker, pigmented skin. For example, among patients presenting with uveitis, about one in ten in Japan and one in 50 in India have VKH syndrome [6,7]. It presents acutely with varying symptoms and signs, which include meningoencephalitis, visual blurring, and deafness. The eye signs are very characteristic and can help to make the diagnosis. The most prominent ocular finding is intensely pink optic nerves (see Figure 1), with severe visual reduction and loss of function, which accounts for the absent pupillary responses.

**Figure 2:**
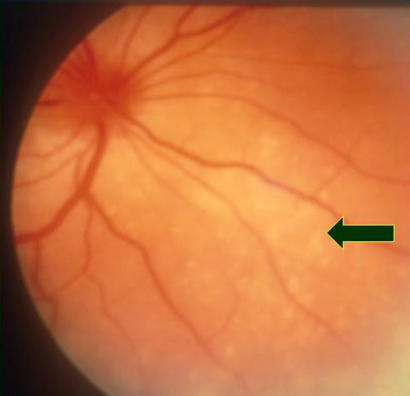
White Choroidal Infiltrates (Arrow) Seen in VKH Syndrome with Very Pink Optic Nerve Head

**Figure 3:**
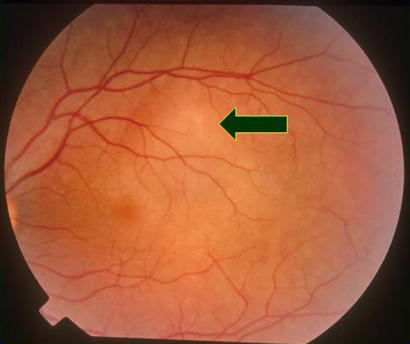
Localised Retinal Detachment

**Figure 4:**
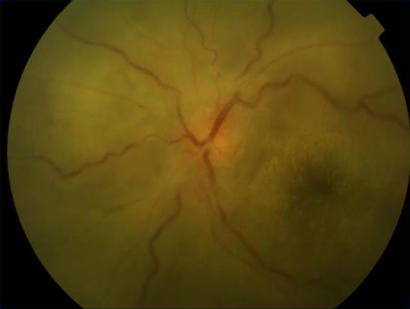
Total Retinal Detachment, Where Whole Retina is Grey in Colour

**Figure 5:**
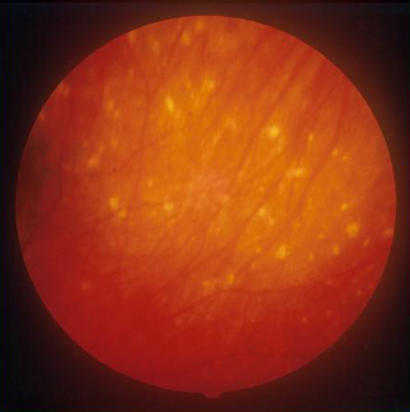
Scarring When Choroidal Granulomas Subside

VKH syndrome is usually bilateral, but occasionally the eyes can be affected asymmetrically so that one is very mildly involved. The syndrome is accompanied by marked intraocular inflammation, and there are choroidal infiltrates (Figure 2) associated with serous retinal detachments, which may be localised (Figure 3) or affect the whole retina (Figure 4). It is likely that these detachments are due to the retinal pigment epithelium (RPE) being affected by the underlying inflammatory choroidal granulomas (which heal leaving scars; see Figure 5), and fluid accumulates underneath the retina because of reduced function of the RPE when it becomes inflamed.

**Figure 6:**
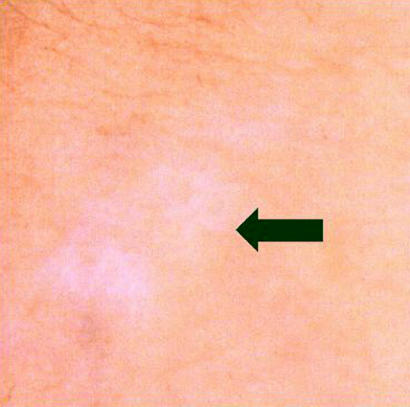
Vitiligo on Skin of Forearm

**Figure 7:**
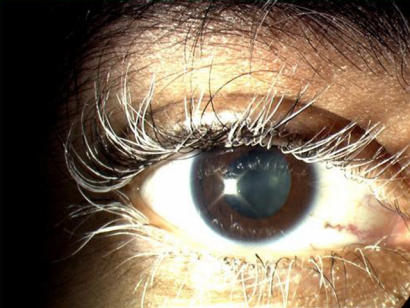
Poliosis Note white eyelashes on child.

The disorder is caused by an immune response to melanin and affects parts of the body where melanin is found. The initiating stimulus for this response is unknown, but T-cells sensitised to melanin-associated antigens are found in the peripheral blood. In the ear, the melanocytes of the inner ear are the target, and the inflammatory response here results in hearing loss and balance problems. In longstanding untreated cases, depigmentation may occur in other sites such as skin (vitiligo; Figure 6) and eyelashes (poliosis; Figure 7), but these are uncommon when corticosteroids and other immunosuppressive agents are used in treatment. Depigmentation of the RPE can also occur, giving a ‘sunset’ appearance to the dark fundus.

Treatment with high-dose corticosteroids is essential [3] and should be initially 1–2 mg/kg/day. This treatment can be given orally or intravenously, depending on how unwell the patient is. However, patients commonly need other immunosuppressive agents as well, so as to allow the dose of steroids to be reduced more quickly. Both cyclosporin and mycophenolate are very useful as steroid-sparing agents, with cyclosporin having the advantage of a variable-dose regimen for more rapid onset of action. On the down side, cyclosporin can cause hirsutism, especially in combination with corticosteroids (which can also cause this side effect). Unfortunately, the costs of cyclosporin and mycophenolate may preclude their use in resource-poor settings, with the result that patients may require high-dose corticosteroids for much longer, with all the concomitant side effects. Inadequate initial treatment may increase the risk of recurrence and long-term complications.

Treatment is required until the disease goes into remission. The meningoence-phalitic signs and retinal and choroidal signs settle quickly, often within a week or so, whereas the optic nerve inflammation may take longer to settle. The visual prognosis depends on the degree of permanent damage to the optic nerve and the macula area, which often shows considerable pigment clumping as a result of the damage to the RPE.

Relapse affecting the optic nerve, choroids, and retina is uncommon, provided that treatment has been given for long enough. However, recurrent anterior uveitis requiring steroid drops is common. This is not a threat to sight if adequately controlled. As with any other cause of intraocular inflammation particularly associated with choroidal involvement, VKH syndrome can lead to reduced vision via cataracts, glaucoma damaging the optic nerve, and new vessels growing into the retina through the damaged RPE (choroidal neovascular membrane).

**Key Learning Points**

- Consider meningitis in the differential diagnosis of a patient presenting with fever and headache.
- CSF analysis is essential to confirm meningitis and as part of establishing the cause.
- Consider non-infectious causes when a patient does not respond rapidly to therapy.
- Blurring of vision must be investigated and may help in determining the underlying diagnosis or the presence of papilloedema.

**Suggested Reading**

SutlasPNUnalAFortaHSenolSKirbasD2003Tuberculous meningitis in adults: Review of 61 casesInfection31387391Early suspicion and appropriate long-term anti-tuberculosis therapy together with corticosteroids may reduce mortality and morbidity in patients with TB meningitis1473538010.1007/s15010-003-3179-1RedingtonJJTylerKL2002Viral infections of the nervous systemArch Neurol59712718This review is an update on diagnosis and treatment1202025010.1001/archneur.59.5.712ThomsonRBJrBertramH2001Laboratory diagnosis of central nervous system infectionsInfect Dis Clin North Am1510471071This paper discusses conventional tests, such as culture, and others such as antigen testing and PCR1178026710.1016/s0891-5520(05)70186-0RotbartHA2000Viral meningitisSemin Neurol20277292The virology, pathogenesis, epidemiology, clinical manifestations, diagnostic studies, and established and potential antiviral therapies for viral meningitis are discussed. A differential diagnosis of the aseptic meningitis syndrome is provided1105129310.1055/s-2000-9427NegriniBKelleherKJWaldER2000Cerebrospinal fluid findings in aseptic versus bacterial meningitisPediatrics105316319Polymorphonuclear cell predominance in the CSF does not discriminate between aseptic and bacterial meningitis1065494810.1542/peds.105.2.316KamondiASzegediAPappASeresASzirmaiI2000Vogt-Koyanagi-Harada disease presenting initially as aseptic meningoencephalitisEur J Neurol7719722This paper describes the neurological and eye signs in VKH syndrome1113636210.1046/j.1468-1331.2000.00156.xSeehusenDAReevesMMFominDA2003Cerebrospinal fluid analysisAm Fam Physician6811031108Lumbar puncture is frequently performed in primary care, and this review outlines the interpretation of the clinical and laboratory findings14524396ShahKHEdlowJA2002Distinguishing traumatic lumbar puncture from true subarachnoid hemorrhageJ Emerg Med236774The purpose of this article is to assist emergency physicians in distinguishing traumatic lumbar punctures from subarachnoid hemorrhage1221747410.1016/s0736-4679(02)00464-x

## Abbreviations

CSF: cerebrospinal fluid
PCR: polymerase
RPE: retinal pigment epithelium
TB: tuberculous
VKH: Vogt-Koyanagi-Harada
WBC: white blood cell
